# Correction: Supramolecular ionogels prepared with bis(amino alcohol)oxamides as gelators: ionic transport and mechanical properties

**DOI:** 10.1039/d0ra90061c

**Published:** 2020-05-27

**Authors:** Ana Šantić, Marc Brinkkötter, Tomislav Portada, Leo Frkanec, Cornelia Cramer, Monika Schönhoff, Andrea Moguš-Milanković

**Affiliations:** Laboratory for Functional Materials, Division of Materials Chemistry, Ruđer Bošković Institute Bijenička c. 54 10000 Zagreb Croatia asantic@irb.hr; Institute of Physical Chemistry, University of Muenster Corrensstraße 28/30 48149 Münster Germany; Laboratory of Supramolecular Chemistry, Division of Organic Chemistry and Biochemistry, Ruđer Bošković Institute Bijenička c. 54 10000 Zagreb Croatia

## Abstract

Correction for ‘Supramolecular ionogels prepared with bis(amino alcohol)oxamides as gelators: ionic transport and mechanical properties’ by Ana Šantić *et al.*, *RSC Adv.*, 2020, **10**, 17070–17078, DOI: 10.1039/D0RA01249A.

The authors regret that the name of one of the authors (Cornelia Cramer) was shown incorrectly in the original article. The corrected author list is as shown above.

The Royal Society of Chemistry apologises for these errors and any consequent inconvenience to authors and readers.

## Supplementary Material

